# Motor cortex gliomas induces microstructural changes of large fiber tracts revealed by TBSS

**DOI:** 10.1038/s41598-020-73746-1

**Published:** 2020-10-09

**Authors:** Xiangdong Wang, Chunyao Zhou, Lei Wang, Yinyan Wang, Tao Jiang

**Affiliations:** 1 Beijing Neurosurgical Institute, Capital Medical University, Beijing, China; 2 Department of Neurosurgery, Beijing Tiantan Hospital, Capital Medical University, No. 119 West South Fourth Ring Road, Beijing, 100070 China; 3 Department of Neurosurgery, Heji Hospital, Changzhi Medical College, Changzhi City, Shanxi Province China

**Keywords:** CNS cancer, White matter disease

## Abstract

Gliomas grow and invade along white matter fiber tracts. This study assessed the effects of motor cortex gliomas on the cerebral white matter fiber bundle skeleton. The motor cortex glioma group included 21 patients, and the control group comprised 14 healthy volunteers. Both groups underwent magnetic resonance imaging-based 3.0 T diffusion tensor imaging. We used tract-based spatial statistics to analyze the characteristics of white matter fiber bundles. The left and right motor cortex glioma groups were analyzed separately from the control group. Results were statistically corrected by the family-wise error rate. Compared with the controls, patients with left motor cortex gliomas exhibited significantly reduced fractional anisotropy and an increased radial diffusivity in the corpus callosum. The alterations in mean diffusivity (MD) and the axial diffusivity (AD) were widely distributed throughout the brain. Furthermore, atlas-based analysis showed elevated MD and AD in the contralateral superior fronto-occipital fasciculus. Motor cortex gliomas significantly affect white matter fiber microstructure proximal to the tumor. The range of affected white matter fibers may extend beyond the tumor-affected area. These changes are primarily related to early stage tumor invasion.

## Introduction

The motor cortex is surrounded by and connected to large white matter fiber bundles consisting of the superior longitudinal fasciculus, superior fronto-occipital fasciculus, pyramidal tracts, and U-shaped fibers^1,2^. The motor cortex is predisposed to gliomas. Approximately 25% of low-grade gliomas and 10% of high-grade gliomas occur in the motor cortex, and most motor cortex gliomas are low-grade. Gliomas of the frontal lobe often grow along the white matter fiber bundles and form complex tumors with finger-like processes. This leads to corresponding structural damage and dysfunction. Accurate preoperative localization of the range of tumor invasion can minimize complications caused by surgery-related cortical or white matter damage, including epilepsy, cognitive dysfunction, and motor dysfunction^3–5^.

Glioma invasion of functional areas may cause localized dysfunction in different regions and may subsequently activate brain plasticity^6–9^. The growth and invasion of brain gliomas are relatively slow processes, during which the brain may structurally and functionally reorganize sites local or distant to the lesion to maintain function^9^. This reorganization may facilitate postoperative recovery. However, the nature of white matter fiber bundle plasticity during motor cortex glioma growth is unclear.

Diffusion tensor imaging (DTI) is an important method to characterize the structure of intracranial white matter. The processing methods to reconstruct diffusion data enable us to understand how white matter fiber tracts are altered in patients with gliomas. Among the post-processed diffusion maps, the fractional anisotropy (FA) map reportedly provides the best indication of fiber degeneration or reorganization status. Recent studies have shown that DTI has higher diagnostic sensitivity to the early invasion of gliomas than standard magnetic resonance imaging (MRI)^10^.

A DTI glioma study revealed significant glioma-related changes in many white matter regions, including changes in the degree of anisotropy, average diffusion coefficient (AD), and other DTI coefficients; however, no conventional MRI study has reported glioma-related signal changes^11,12^. These changes identified by DTI may indicate the invasion and spread of early tumor cell precursors that may not be detected with conventional MRI^13^. However, previous studies have typically focused on the analysis of FA dispersion values and related issues of one or more white matter fiber pathways, but overall have lacked voxel-level assessments. DTI is a highly efficient, non-invasive technique that allows in vivo analysis of neuroplasticity of macroscopic brain connections^14–17^. However, there are limited DTI research studies into the changes in subcortical fiber bundle plasticity caused by motor cortex gliomas.

Tract-based spatial statistical (TBSS) analysis of whole-brain fiber bundles^18^ enables statistical assessment of variations in the major white matter pathways of the whole brain at the voxel level. The accuracy and reproducibility TBSS analyses are superior to those of conventional fiber bundle tracking or whole-brain voxel-level analyses. The current study assessed the effects of motor cortex glioma growth on white matter fiber bundles using DTI and TBSS techniques. These changes in white matter fiber bundles may be precursors to brain function compensatory mechanisms or other changes. The results of this study may elucidate the mechanisms of early invasion in patients with motor cortex gliomas.

## Methods

This study was approved by the Institutional Review Broad of Beijing Tiantan Hospital. Written informed consent was obtained from every enrolled participant among both the patient and control groups. All methods were carried out in accordance with relevant guidelines and regulations.

### Participants

Thirty patients (15 males, 15 females; age range: 19–66 years; mean ± SD, 44.17 ± 10.42 years) who were diagnosed with motor cortex gliomas at our hospital between 2014 and 2019 were originally recruited. Of the 30 patients, 8 were excluded based on contralateral involvement on T2-weighted images. One patient who was significantly younger than the others was also excluded. Finally, 21 patients (12 males, 9 females; age range: 30–64 years; mean ± SD = 43.23 ± 10.60 years) with left motor cortex gliomas were included in the final analysis. Fourteen age and gender-matched healthy controls were also enrolled(7 males, age range: 41–50 years; mean ± SD = 45.21 ± 4.3 years). All subjects underwent conventional MRI and DTI. Basic clinical data of the patients were assessed. All patients underwent tumor resection and were pathologically diagnosed with grades II–IV gliomas, according to the World Health Organization (WHO) 2016 classification of central nervous system tumors.

### MRI data acquisition

A MAGNETOM Prisma 3.0 T scanner (Siemens; Erlangen, Germany) was used to perform conventional anatomical MRI with the following scanning parameters: T1-magnetization prepared rapid acquisition gradient echo was applied to collect anatomical images (repetition time, TR: 2300 ms; echo time, TE: 2.3 ms; flip angle: 8°; field of view, FOV: 240 × 240 mm^2^; voxel size: 1.0 × 1.0 × 1.0 mm^3^; slice number: 192). The tumor location was determined by two board-certified neurosurgeons and confirmed by an experienced neuroradiologist, all of whom were blinded to the clinical information of the patients. The DTI used a single-shot, echo-planar imaging (EPI) sequence as follows: axial slices = 75; resolution = 2.0 × 2.0 × 2.0 mm; TR = 6000 ms; TE = 103 ms; FOV = 230 × 230 mm, 30 different directions; b = 0/1000 s/mm^2^; EPI factor = 154.

### Tumor region of interest (ROI) extraction

The tumor regions of each patient were manually segmented on T2-weighted images by two experienced neurosurgeons, using MRIcron software (https://www.nitrc.org/projects/mricron). Abnormally hyperintense signals on T2-weighted images were identified as tumor areas. Areas containing cerebrospinal fluid were carefully avoided, as they may cause mis-masking of tumor margins. The lesion map was re-evaluated by an experienced neuroradiologist, and if the discrepancy was > 5%, the final determination was made by the radiologist. All extracted tumor masks were then co-registered to the diffusion space for further masking and processing of DTI data. All normalized tumor masks were normalized into the Montreal Neurological Institute standard space using the clinical toolbox package in Statistical Parametric Mapping (SPM8) (https://www.fil.ion.ucl.ac.uk/spm/software/spm8/). A tumor overlapping image was also created with all normalized tumor masks using MRIcron software.

### Diffusion data processing

Data preprocessing and statistical analysis were performed using the pipeline toolbox, PANDA (https://www.nitrc.org/projects/panda/), which was developed based on the FMRIB software library (FSL; https://fsl.fmrib.ox.ac.uk/fsl/fslwiki/). Data processing steps and TBSS analysis were previously described by Cui^19^. Briefly, steps to extract basic DTI metrics were implemented, including extracting a brain mask, correcting for eddy current effects, averaging multiple acquisitions, calculating diffusion tensors, and producing metrics. The TBSS process was then conducted, which included alignment of each subject’s masked FA image, creation of the mean FA map, extraction of the FA skeleton, and projection of each individual subject’s FA image onto the skeleton^18,20^. Moreover, masks of every fiber bundle in the ICBM-DTI-152 atlas were extracted to obtain the mean FA, MD, AD, and RD values within those regions. For each patient or control, a total of 50 mean values in the fiber bundles of each DTI metric were generated.

### Statistical analysis

Voxel-wise analysis was conducted, using a general linear model via the FSL randomize tool^21^, which consisted of a 5000-repetition permutation test between the glioma and healthy control groups. Significant clusters were defined using the threshold-free cluster enhancement method^20^, and the acquired p-value maps were further corrected by family-wise error (FWE) at the threshold-free cluster enhancement (TFCE) level at *p* < 0.05. The cluster-locator tool was used to locate significant clusters in specific fiber tracts in the ICBM-DTI-152 white matter atlas. Only clusters with a voxel size > 100 were considered. The mean DTI metrics extracted by ICBM-DTI-152 fiber masks were also compared between the patients and controls using unpaired *t*-tests and corrected by FWE at a significance level of 0.05. In the statistical analysis, the tumor-involved regions were eliminated from the calculation based on the tumor overlapping map.

## Results

### Clinical characteristics

**Figure 1:**
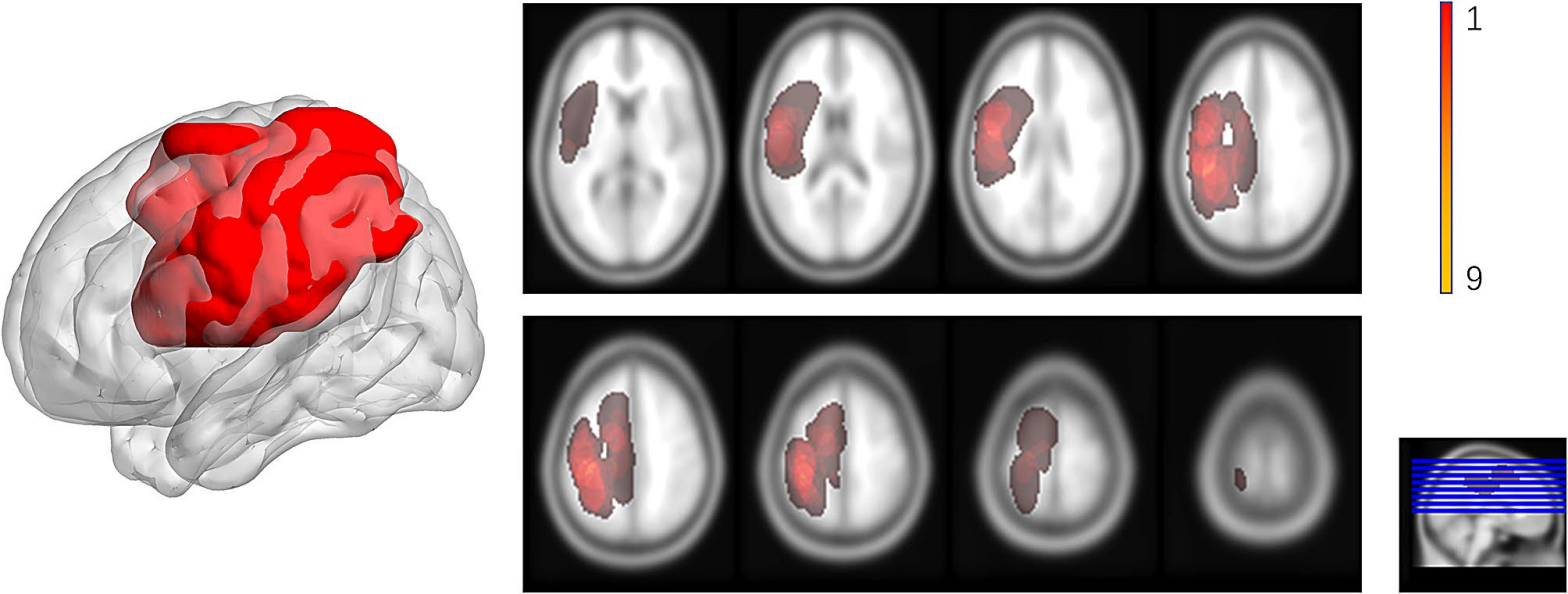
Overlap of all tumor lesions with 3-D rendering of the binary overlapping mask. Voxel color indicates the number of overlapping cases from 1 (red) to 12(orange yellow), which is shown in the color bar. Image is generated by BrainNetViewer V1.53(https://www.nitrc.org/projects/bnv/) and MRIcron version 4 (www.mricro.com).

The clinical information of all enrolled patients is summarized in Table 1. Briefly, 12 males and 9 females were evaluated. There were no significant differences between patients and controls in age (*p* = 0.498, t-test) and gender (*p* = 0.678, chi-squre test). Seventeen lower-grade gliomas (WHO grades II–III) and four glioblastomas (WHO grade IV) were detected. In total, ten patients had preoperative limb weakness, and two experienced cognitive defects (one had memory deterioration and one had slow reactions). Seven patients had a history of tumor-related postsurgical seizures. Two experienced no presurgical symptoms. All tumor locations were close, with fine overlapping. (Fig. 1) Average tumor volume is 28.3 ± 12.25 mm^3^. (Table 1).

**Table 1:** Summary of the patients’ characteristic.

Patient no.	Age (years)	gender	Histopathological type	Preoperative symptoms	Tumor volume (mm^3^)
1	36	M	Anaplastic oligoastrocytoma, WHO grade III	Seizures	28.6
2	52	M	Glioblastoma, WHO grade IV	Seizures	19.7
3	32	F	Anaplastic astrocytoma, WHO grade III	Seizures	44.8
4	42	M	Anaplastic oligoastrocytoma, WHO grade III	Right limb weakness	19.7
5	31	F	Anaplastic astrocytoma, WHO grade III-IV	Seizures	47.1
6	32	M	Astrocytoma, WHO grade II	Seizures	16.1
7	30	M	Anaplastic astrocytoma, WHO grade III	Seizures	32.2
8	43	F	Oligodendroglioma, WHO grade II	Seizures	21.4
9	53	M	Glioblastoma, WHO grade IV	Slow in reacting, disorientation	45.9
10	53	M	Anaplastic astrocytoma WHO grade III	Right limb weakness	38.1
11	41	F	Oligoastrocytoma, WHO gradeII- III	Right limb weakness	15.4
12	57	M	Anaplastic astrocytoma, WHO grade III	Right limb weakness	20.9
13	64	F	Glioblastoma, WHO grade IV	Right limb weakness	18.1
14	30	M	Anaplastic astrocytoma, WHO grade III	Right limb weakness	12.5
15	34	F	Glioblastoma, WHO grade IV	Slow in reacting	18.8
16	52	F	Oligodendroglioma, WHO grade II	Right limb weakness	49.3
17	53	M	Anaplastic astrocytoma, WHO grade III	Right limb weakness	17.6
18	44	M	Anaplastic astrocytoma, WHO grade III	None	18.8
19	35	F	Oligodendroglioma, WHO grade II	None	29.2
20	54	M	Astrocytoma, WHO grade II	Right limb weakness	43.4
21	40	F	Astrocytoma, WHO grade II	Right limb weakness	36.7

F—female, M—male.

### Atlas-based analysis

**Figure 2:**
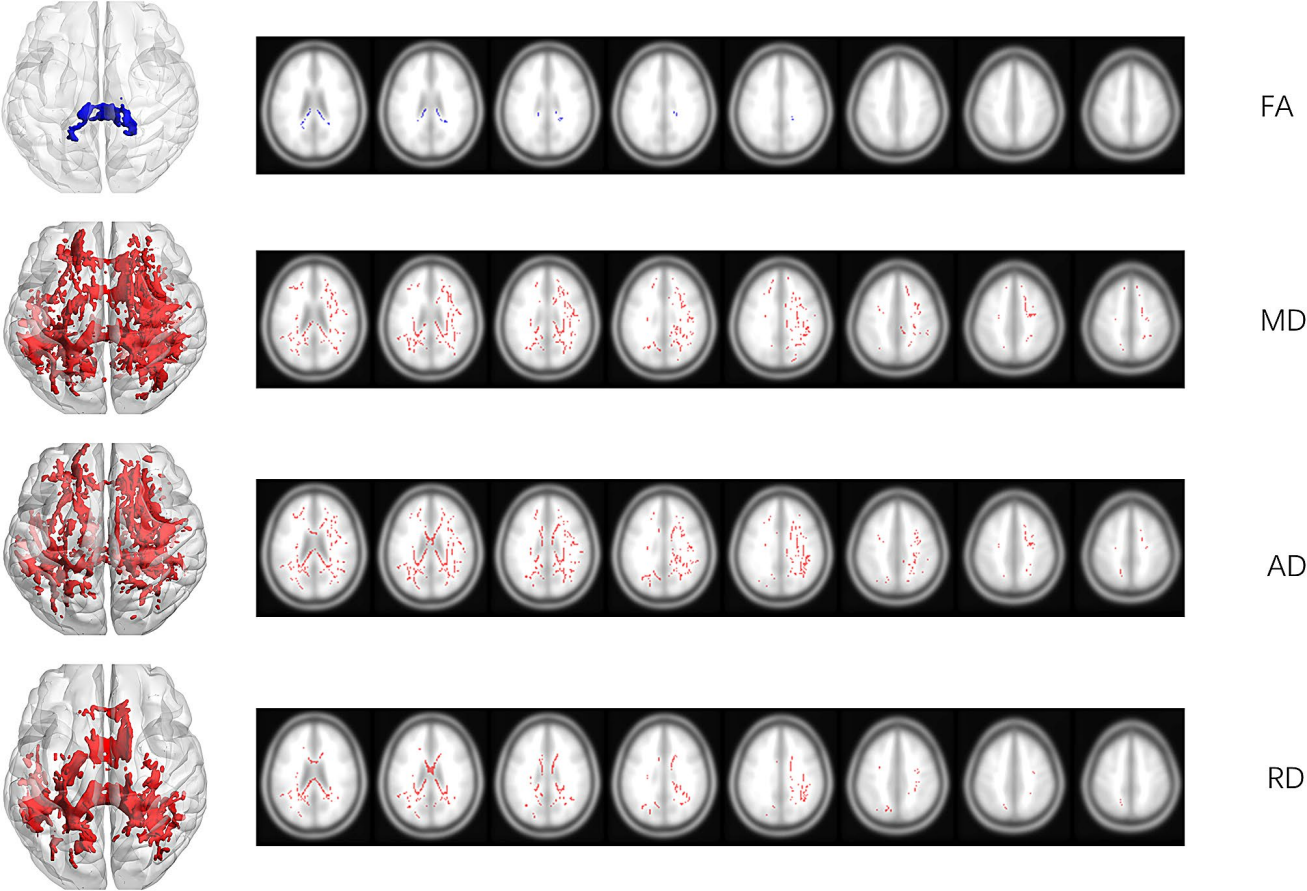
Significant clusters identified by permutation testing (n = 5000, FWE-corrected at TFCE level *p* < 0.05). 3-D rendered figures are shown on the left, and axonal overlapping figures of major crossovers are shown on the right. Red indicates higher values in glioma patients vs. controls, and the blue indicates higher values in control versus glioma patients. Image is generated by BrainNetViewer V1.53(https://www.nitrc.org/projects/bnv/) and MRIcron version 4 (www.mricro.com).

**Figure 3:**
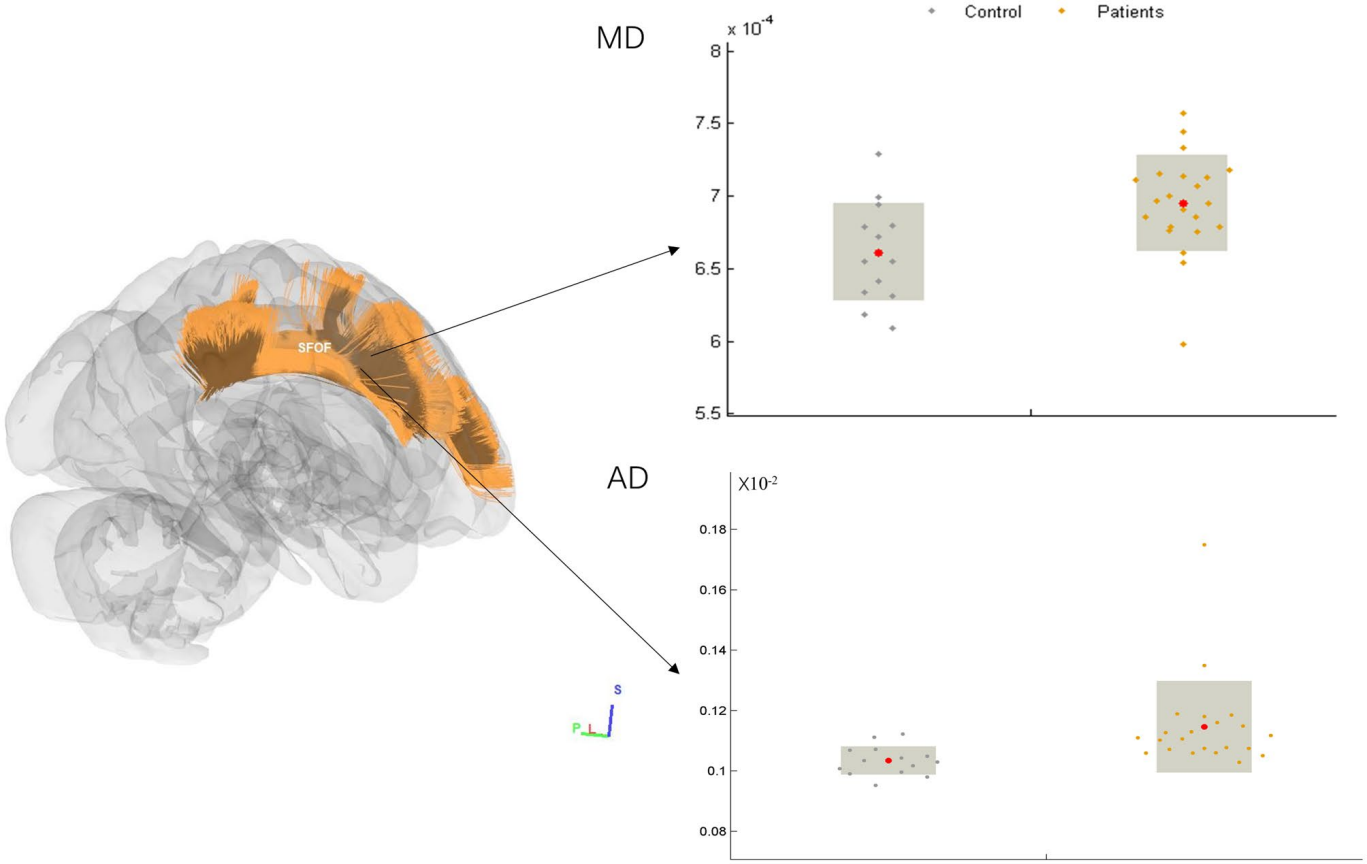
Atlas-based analysis showed significant MD and AD increases in the right superior frontal-occipital fascilicus of glioma patients (T-test, FWE-corrected *p* < 0.05). A dot-bar figure of group values for MD and AD is shown on the left. Grey dots represent the averaged MD or RD values of controls, while orange dots represent those of patients. 3-D rendering image is generated by BrainNetViewer V1.53 (https://www.nitrc.org/projects/bnv/), the dot-bar figure is generated by GRETNA v2.0.0 (https://www.nitrc.org/projects/gretna/).

The inter-group comparisons of mean FA, MD, AD, and RD values of every fiber bundle in the ICBM-DTI-152 white matter atlas showed many significant differences; however, many of the fiber masks showed total or partial overlap with the tumor mask (Fig. 2). The results for such fibers might therefore be inaccurate, owing to the tumor mass effect. After, these were excluded, only the right superior fronto-occipital fasciculus showed significantly elevated MD (FWE-corrected *p* = 0.029) and AD (FWE-corrected *p* = 0.017). No significant differences were detected in FA or RD (Fig. 3).

### Voxel wise analysis

The voxel-wise inter-group comparisons of FA, MD, AD and RD values showed robust differences at the location of several major fiber tracts. Briefly, the major affected fibers were body and genu of corpus callosum, with decreased FA and increased AD,MD and RD in a relatively large voxel count. Furthermore, the left and right sided superior longitudinal fasciculus had increased AD, MD and RD in big voxel count. (Table 2). Also, several other fiber tracts were detected to have significant clusters in one or two diffusion metrics.

**Table 2:** Significant clusters with most voxel count of all diffusion metrics.

Cluster location	Diffusion Metrics			
	FA	MD	AD	RD
Splenium of corpus callosum	− 785	2250	1195	2125
Body of corpus callosum	− 134	1542	445	1492
Genu of corpus callosum	0	1206	822	570
Superior longitudinal fasciculus R	0	1481	1232	696
Superior longitudinal fasciculus L	0	485	404	470

The numbers represents total voxel count of the cluster, negative sign indicates values in the patients group is smaller that that of the controls, positive sign indicates the opposite.

### FA alterations in patients with left motor gliomas

Compared with controls, the patient group showed degenerative alterations in the corpus callosum, as evidenced by reduced FA (Fig. 2). Specifically, one cluster showed reduced FA (voxel size = 1018, corrected *p* < 0.05) in the body of the corpus callosum (134 voxels) and the splenium of the corpus callosum (785 voxels). The entire spatial distribution of altered fiber bundles is presented in Supplementary Table 1.

### MD alterations in patients with left motor gliomas

Global MD elevation was observed in glioma patients compared with controls (Fig. 2). The major significant cluster (voxel size = 33,533) comprised multiple parts of fiber bundles, and fibers with the highest voxel counts were those of the splenium of the corpus callosum (2250 voxels), body of the corpus callosum (1542 voxels), right superior longitudinal fasciculus (1481 voxels), genu of the corpus callosum (1206 voxels). The entire spatial distribution of altered fiber bundles is presented in Supplementary Table 2.

### AD alterations in patients with left motor gliomas

Global AD was observed in comparisons of glioma patients with the controls (Fig. 2). The major significant cluster (voxel size = 29,171) comprised multiple parts of fiber bundles. The fibers with the highest voxel counts were the right anterior corona radiata (1376 voxels), splenium of the corpus callosum (1195 voxels), right superior corona radiata (1162 voxels), and genu of the corpus callosum (822 voxels). The entire spatial distribution of altered fiber bundles is presented in Supplementary Table 3.

### RD alterations in patients with left motor gliomas

Global RD elevation was observed in glioma patients compared to controls (Fig. 2). The major significant cluster (voxel size = 13,297) comprised multiple parts of fiber bundles. Fibers with the highest voxel counts were those of the splenium of the corpus callosum (2125 voxels), body of the corpus callosum (1492 voxels), right superior longitudinal fasciculus (696 voxels), left posterior limb of the internal capsule (648 voxels), and the genu of the corpus callosum (570 voxels). The entire spatial distribution of altered fiber bundles is presented in Supplementary Table 4.

## Discussion

In the present study, we used atlas-based diffusion analysis and TBSS to analyze differences in cerebral white matter fiber bundle characteristics between patients with motor cortex gliomas and normal controls. This approach revealed the influence of motor cortex gliomas on the cerebral white matter fiber bundle skeleton and the subsequent plasticity of white matter fiber bundles.

Motor cortex gliomas are common tumors of the central nervous system. Owing to the specific anatomical location of the frontal lobe, the relationship between motor cortex gliomas and the surrounding fiber bundles is more complicated than for other intracranial tumors. Previous investigations^13–15^ focused on the qualitative analysis of fiber bundles that may be involved in motor cortex tumors using traditional imaging data. These studies were only able to assess changes in the surrounding fibers due to invasion or edema. Such fibers cannot be remodeled to preserve local function, and plastic changes in the white matter typically occur distal to the tumor.

In the present study, TBSS analysis at the voxel level was used to identify fiber bundles that may be involved in motor cortex gliomas. Significant changes were noted in the diffusion coefficients of multiple white matter fiber pathways in the white matter skeleton of the motor cortex glioma group. These changes were predominantly distributed among the main fibers proximal to and connected to the motor cortex. The affected white matter areas were consistent among patients with tumors on either side; however, more extensive changes were noted in patients with right-sided glioma. These white matter fiber bundle changes provide direct evidence for the plasticity of subcortical white matter pathways in response to tumor growth.

White matter fiber bundles represent the structural body of brain connections and form the anatomical basis for glioma invasion. The glioma growth process involves invasion and structural alteration of the surrounding white matter fiber bundles. Changes in the white matter skeleton diffusion coefficient of the ipsilateral cerebral hemisphere are primarily caused by erosion, destruction, impingement, and peritumoral edema of the surrounding fiber bundle due to the tumor itself^22^.

Altered white matter skeleton diffusion coefficients are also important indicators of changes associated with white matter fiber bundle plasticity. As a voxel-level diffusion data analysis method^18^, TBSS can detect slight changes in distant white matter skeletons and eliminate the influence of non-white matter areas on the statistical analysis results. This method of analyzing diffusion data is more accurate than traditional voxel-level approaches^22,23^.

Changes in FA values reflect alterations in subcortical white matter fiber bundles and may indicate early stage tumor invasion. The altered fiber bundles could be in, near, or at remote sites of the tumor. In the present study, the main fiber bundles affected were the splenium and genu of the corpus callosum. We observed reduced FA and elevated MD, AD, and RD values. The affected regions extended to the contralateral hemisphere, indicating that white matter integrity was compromised. This may be a sign of contralateral invasion.

A previous study on insular lobe invasive patterns of low-grade gliomas suggested that they mostly infiltrate alongside long fiber bundles^24^. Interestingly, studies on frontal lobe gliomas that invade the corpus callosum identified elevated MD as a sign of early invasion based on T2-weighted fluid-attenuated inversion recovery sequences^10,11^. Furthermore, large fiber tracts, such as the superior longitudinal fasciculus and superior fronto-occipital fasciculus, exhibited elevated MD, AD, and RD These fibers were all directly connected to the area of the tumor; however, parts of those fibers were eliminated from the analysis because of the mass effect. The increased diffusion metrics in those fibers may have been simply due to slight edema or ischemic degeneration caused by the physical pressure of the tumor, and the unchanged FA indicated intact and undisrupted fiber structures. As a result, those fibers were less likely to be invaded by tumor cells.

In our atlas-based analysis, only the contralateral superior fronto-occipital fasciculus showed significantly elevated MD and AD, which is a strong indication of invasion potential to the contralateral hemisphere. Given this structure’s proximity to the corpus callosum, alterations in this tract might be regarded as an extension of the changes in the corpus callosum. Interestingly, the atlas-based analysis showed results that differed from those of the voxel-level analysis. A major cause of this discrepancy is difference in algorithm. In the voxel-wise inter-group comparison, the system would first extract diffusion values of every subject in the voxel at the same coordinate, and then present a voxel-by-voxel permutation test, and finally perform the TFCE-based correction. Thus, the significant region would be a collection of auto-defined clusters, and the next step would be to identify the fibers where the specific clusters were located.

Notably, when we discovered large clusters within a specific fiber bundle, it did not necessarily indicate that the entire fiber showed an inter-group difference. However, in the atlas-based analysis, the system first evaluated the mean value of all voxels within the fiber ROI (as defined by the atlas), and we performed an inter-group comparison based on those ROI-based average values. The results were therefore based on entire fiber tracts.

## Conclusion

In this study, TBSS was used to quantitatively assess white matter changes in large fiber bundles, which may be caused by motor cortex glioma growth and invasion. Fiber bundles that exhibited significantly altered diffusion coefficients were primarily those of the corpus callosum; the coefficient changes included decreased FA and increased MD, AD, and RD. These changes in white matter fiber bundles may be precursors of glioma invasion.

### Limitations

The current research used TBSS to explore microstructural white matter alterations associated with motor area glioma. The results revealed a potential invasive pattern of tumors; however, there are some limitations to be considered. Due to technological reasons, we only used DTI for analysis, so structural and functional inter-group differences in the cortex may have been missed. The study design did not include quantitative analyses of specific neurological deficits such as cognitive score and muscle strength grading. We were unable to carry out a voxel-wise analysis considering functional deficits and specific white matter structure for the current dataset. The number of enrolled patients was too small to compare subgroups, such as patients with different tumor grades, with or without limbal weakness, or with or without cognitive damage. Our future research will focus on expanding the dataset and gathering quantitative scores. With larger patient numbers, cognitive test results, and other imaging data such as functional MRI and arterial spin labeling, further multi-model analyses with connectome construction and graph-theory analyses could clarify the effects of motor area gliomas.

## Supplementary information

Supplementary Information 1.

Supplementary Information 2.

Supplementary Information 3.

Supplementary Information 4.

## Data Availability

All clinical data of the current study is available if requested.

## Abbreviations

FA: Fractional anisotropy
MD: Mean diffusivity
AD: Axial diffusivity
RD: Radial diffusivity
TBSS: Tract based spacial statistics
TFCE: Threshold free cluster enhancement
FDR: False discovery rate
FWE: Family wise error
